# Enhancing Parent–Child Interaction and Self-Efficacy in Motor Skills Development for Young Children with Developmental Delays

**DOI:** 10.3390/children13020309

**Published:** 2026-02-23

**Authors:** Yu-Lin Lai, Szu-Yin Chu, I-Huei Lee, Hsiu-Wen Yang

**Affiliations:** 1 Department of Physical Medicine and Rehabilitation, National Taiwan University Hospital Hsin-Chu Branch, Hsinchu 300197, Taiwan; 2 Department of Special Education, National Tsing Hua University, Hsinchu 300044, Taiwan; ihlee@mx.nthu.edu.tw; 3 Frank Porter Graham Child Development Institute, University of North Carolina at Chapel Hill, Chapel Hill, NC 27599, USA; hsiu-wen.yang@unc.edu

**Keywords:** children with developmental delays, parent participation, home program, parent–child interaction, parental self-efficacy

## Abstract

**Highlights:**

**What are the main findings?**

- Child goal achievement and parental outcomes improved: After the 8-week home program, children showed significant improvements in Goal Attainment Scale (GAS) scores, indicating successful achievement of targeted goals, while parents demonstrated significant gains in parenting competence, confidence, and overall parental self-efficacy.
- Parent–child interaction did not significantly change: No significant improvement was found in parent–child interaction.

**What is the implication of the main finding?**

- Strengthen family-centered, goal-focused home programs: Clinicians should actively involve parents in goal setting using tools such as the GAS and collaborate with families to design home programs that are feasible, motivating, and integrated into daily routines, as parental participation meaningfully supports children’s developmental goal achievement.
- Build parental self-efficacy through coaching and partnership: Professionals should prioritize parent coaching, motivational strategies, and ongoing communication to enhance parents’ confidence and competence in guiding their children, as increased parental self-efficacy can improve adherence to home programs and ultimately enhance therapeutic outcomes.

**Abstract:**

**Background/Objectives**: The present study investigated the effects of parental participation in home program intervention on parent–child interactions, parental self-efficacy, and the goal attainment of children with developmental delays in motor skills. **Methods**: While the interviews consisted of qualitative data, quantitative analyses were applied to the results, making this a mixed-methods study. Participants were 2–6-year-old young children and their families. Twenty-three parent–child dyads were randomly assigned to an intervention group (n = 13) or a comparison group (n = 10). Outcomes were evaluated using the Parent–Child Interaction Questionnaire and Parental Self-Efficacy Questionnaire. **Results**: Improvements in parental self-efficacy and in the Goal Attainment Scale scores of the children were evident in the posttest, whereas no evidence of differences in improvement was found in parent–child interactions between the intervention and comparison groups on the pretest and posttest. **Conclusions**: Parent collaboration with therapists has a significant impact on achieving functional goals for young children, and parental involvement in intervention programs effectively enhances parental self-efficacy.

## 1. Introduction

In Taiwan, the concept of “family-centered” intervention has gradually gained prominence. The goal of early intervention is to provide family support, with a focus on family strengths. Empowering parents and improving family functions can enhance the therapeutic effectiveness for children with developmental delays [1,2]. Within this family-centered framework, the theoretical basis for the development of early interventions for children with developmental delays extends beyond neurobiology, behavioral education, and psychology to include family function [3,4]. Accordingly, parent–child interaction and overall family functioning are increasingly recognized as critical factors influencing intervention outcomes [5,6].

From the ecological system perspective, the family is the “microsystem” that is most closely related to the child. The resources and capabilities of the family affect access to and the effectiveness of interventions for children with developmental delays [7,8,9]. Studies have demonstrated that the most crucial influence on preschool children with developmental delays is the family [4,10]. To enhance the effectiveness of early intervention for children with developmental delays, the family as a whole must be included in intervention programs, and resources should be integrated to enhance family function and provide holistic care for young children [5,6].

Family-centered service involves service providers demonstrating respect, trust, and acceptance when they communicate and coordinate with the families of children with developmental delays [7,11,12]. Empowering parents and improving family function to promote the development of children through teamwork are essential elements of a successful intervention program [7,11,13,14]. Family-centered services can improve parenting satisfaction, improve parental self-efficacy and competence, and reduce parenting stress, ultimately improving the development and quality of life of children with developmental delays [8,15,16].

Parents with high parenting effectiveness are more likely to exhibit positive parenting behaviors [17,18,19,20,21], which are closely related to parental self-efficacy and participation. In children with developmental delays, higher parental self-efficacy is associated with greater involvement in interventions, supporting better motor and adaptive outcomes [22,23,24]. For instance, parent-focused interventions for children with recent diagnoses, such as autism spectrum disorder, have been shown to enhance parental self-efficacy and participation, ultimately benefiting children’s development [25].

Beyond structured parent-training programs, many early intervention services rely heavily on home-based activities, particularly in the domain of motor skill development, where repeated practice in natural environments is essential. Motor skills form the foundation for children’s participation in daily activities and learning and are therefore a core target of early intervention for children with developmental delays. However, motor-focused home programs have traditionally prioritized child motor outcomes, with less attention given to relational processes occurring during parent–child interactions. As a result, the impact of motor-based home interventions on parent–child interaction quality remains insufficiently examined, despite parents playing a central role in implementing these programs.

### Parent–Child Interaction Theratical Framework

Parent–child interaction refers to the process of interaction between parents and children in the natural environment of the family and encompasses parenting behaviors, conversations, internal psychological feelings, and specific behavioral contact [14]. Attachment theory, proposed by Ainsworth in 1979, describes a secure attachment as the basis for trust between an infant and mother, enabling family functioning and supporting children’s potential and social development [26]. Bronfenbrenner’s ecological systems theory, proposed in the late 1970s, emphasizes how interactions within and between systems affect individuals, with the microsystem, such as home and school, being foundational in early childhood development [21]. Parent–child interaction is a key driver of children’s learning and development.

Because parent–child interaction significantly affects young children’s development, many studies have investigated it. A large meta-analysis of 102 parenting RCTs in the first 3 years of life found parenting interventions (mostly home- or home-plus-clinic based) produced moderate improvements in parent–child interactions across low-, middle-, and high-income countries [27]. A systematic review of 17 home-visiting parenting programs reported that 77% of studies improved mother–infant interaction, mainly by increasing sensitivity and responsiveness [28]. Effects were stronger when programs explicitly taught responsive caregiving. Booth-LaForce et al. [29] explored the Promoting First Relationships program, which improved the quality and responsiveness of parent–child interactions and enhanced caregivers’ understanding of children’s emotions. Meijssen et al. [30] designed the home-based Infant Behavioral Assessment and Intervention Program for preterm infants, finding that its family-centered approach promoted sensitive mother–infant interactions at home. Kristensen et al. [31] implemented a video feedback intervention for first-time mothers, which strengthened parent–infant relationships and improved maternal psychosocial functioning.

However, most of this work has focused on relationally oriented programs, rather than interventions in which motor development is the primary target. Consequently, there remains a gap in understanding whether and how parent–child interaction changes when parents participate in motor-focused home programs, particularly within family-centered early intervention models and in Taiwanese contexts. Therefore, the present study investigated the following questions:

(1) How does parental participation in a home program intervention affect the goal attainment of children with motor developmental delays?

(2) How does parental participation in a home program intervention affect parent–child interactions for children with motor development delays?

(3) How does parental participation in a home program intervention for children with motor development delays affect parental self-efficacy?

## 2. Methods

### 2.1. Research Procedure

**Figure 1:**
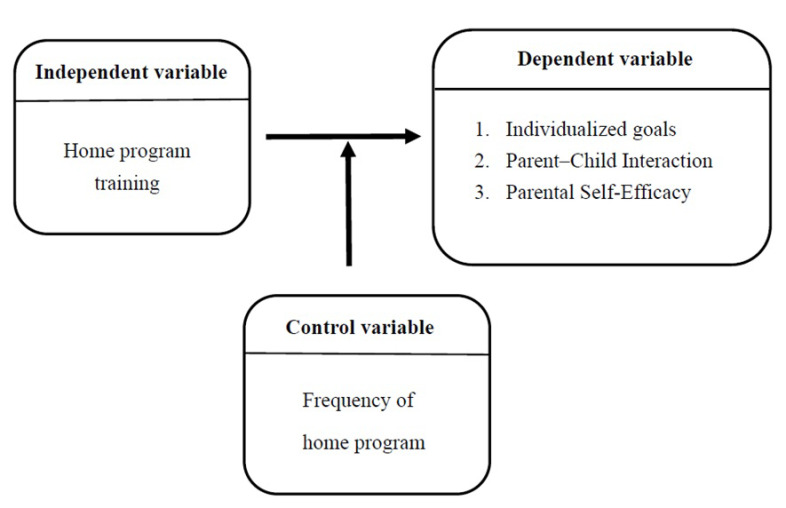
Research Framework.

This study was conducted during the COVID-19 pandemic, during which it was not feasible to control all variables as strictly as in an experimental design. The study included an intervention group and a comparison group to examine the effects of parental participation at home on motor skill development in children with developmental delays, with particular attention to parent–child interaction, parental self-efficacy, and children’s Goal Attainment Scale (GAS) scores. The overall research framework is illustrated in Figure 1.

After providing informed consent, participants were randomly assigned to either the intervention group or the comparison group using a random number table, and parents were blinded to group allocation. Parents were unaware of their own group assignment and did not know the content in which the other group was participating. Prior to the intervention, all parents completed the Parent–Child Interaction Questionnaire and the Parental Self-Efficacy Questionnaire. During the 8-week intervention period, parents in the intervention group were instructed to implement the home program for at least 30 min per day, five days per week. In contrast, parents in the comparison group received general education regarding the home program from case therapists following each treatment session, consistent with usual practice. All children continued to receive their regular early intervention treatment throughout the 8-week period. After the intervention, parents in both groups again completed the Parent–Child Interaction Questionnaire and the Parental Self-Efficacy Questionnaire.

### 2.2. Independent Variable

The independent variable in this study was the home program. After the participants’ basic information was collected and they completed the Parent–Child Interaction Questionnaire and the Parental Self-Efficacy Questionnaire, the intervention group and comparison group were separately briefed on the home program intervention plan through different methods. In the intervention group, the primary therapists of the children collaborated with the children’s parents to establish three individualized goals for the children and designed a home program with consideration of each child’s home environment. The comparison group continued to participate in the original therapeutic program, and the therapists advised parents on implementing the home program in accordance with routine practice. Subsequently, the parents in the intervention group were asked to implement the home program intervention for 8 weeks.

### 2.3. Dependent Variables

The dependent variables in this study were the individualized goals for the children, parent–child interaction, and parental self-efficacy. The current study investigated the changes in these dependent variables after the parents participated in the home program interventions for children with motor developmental delays.

### 2.4. Control Variable

The control variable in this study was the frequency of the home program. Parents in the intervention group were requested to implement the home program for at least 30 min once a day, 5 days a week, for an intervention period of 8 weeks. The objectives were reviewed every 4 weeks for progress. Parents were asked each week to record the implementation of home exercises, and consultation was provided to address any difficulties encountered. The comparison group received general advice on the home program from case therapists during weekly post-treatment consultations, without specific instructions regarding the amount or frequency of home practice. As a result, the extent of home practice was not standardized across groups and was therefore not treated as a controlled variable in the present study.

### 2.5. Participants

Participants were recruited from an early intervention center in a hospital in Taiwan with consideration of the following: the children were between 2 and 6 years of age, had been given a diagnosis of motor developmental delay, were receiving early intervention, and were living with their parents. Children whose main needs did not involve physical disabilities or who had caregivers who could not actively engage in the home program or were unable to complete the Chinese scales by themselves were not included in this study.

### 2.6. Measures

#### 2.6.1. Goal Attainment Scale

The Goal Attainment Scale (see Supplementary S3) was selected as the tool for evaluating goal setting in the intervention group. The Goal Attainment Scale was developed by King et al. [32] and is mainly used to assess the extent of children’s progress in school and to set individual goals for children. Each goal is measured using a 5-point scale, with “−2” indicating current performance, “0” indicating expected performance, and “+2” indicating the best possible performance. Three individualized goals were set for each participant by the children’s therapists, who collaborated with the parents at the beginning, during the goal-setting process, and the three goals were reviewed every 4 weeks to track the children’s progress. Each therapist reviewed Sun [33] prior to discussing GAS with parents, in order to ensure consistency and shared consensus among therapists regarding the use of GAS.

#### 2.6.2. Parent–Child Interaction Questionnaire

The researcher developed the Parent–Child Interaction Scale through a review of the relevant literature and by referencing the Parent–Child Interaction Scale created by Yang [34]. Upon initial draft completion, four experts with experience in the field of parenting were commissioned to review the content of the scale to determine the content validity. The four experts included senior early intervention clinical practitioners from the hospital and a professor from the special education department. The experts assessed the scale on the basis of the initial design of this study by using the ratings of Highly Appropriate, Appropriate, Not Very Appropriate, and Not Appropriate at All, which were assigned scores of 4, 3, 2, and 1, respectively. The content validity index (CVI) was calculated for each item. Items with a CVI greater than or equal to 0.75 were retained for modification, and items with a CVI less than 0.5 were removed. The questionnaire was used to analyze three aspects, namely psychological interaction, behavioral interaction, and verbal interaction. Psychological interaction was measured using 10 survey items, including “I always give positive responses to my child when they perform well”. Behavioral interaction was measured using eight survey items, including “I usually play with toys or play games with my child”. Verbal interaction was measured using nine survey items, including “I usually chat with my child, and we share information about our daily experiences”. Scoring was completed using a 5-point Likert scale, and the overall maximum score for the parent–child interaction scale was 135 points. The higher the score was, the higher the parents’ evaluation of parent–child interaction was. The Parent–Child Interaction Questionnaire was completed before the intervention and after 8 weeks of the intervention.

#### 2.6.3. Parental Self-Efficacy Questionnaire

The researcher referred to the First-Time Mother Parenting Self-Efficacy Scale created by Liu [35] to develop the Parental Self-Efficacy Questionnaire, which was then revised by the same four experts who worked on the Parent–Child Interaction Scale to further establish the content validity. The questionnaire was divided into three parts, namely parenting competence, parenting confidence, and self-evaluation of parenting role. Parenting competence was measured using eight survey items, including “I am very aware of my child’s daily routine”. Parenting confidence was reverse-coded and measured using seven survey items, including “I feel anxious about whether I know how to raise a child”. Self-evaluation of parenting role was measured using six survey items, including “Overall, I feel like I am a good parent”. A 5-point Likert scale was used for scoring, and the overall maximum parenting efficiency score was 105 points. The higher the score was, the higher the overall parental self-efficacy was. The Parental Self-Efficacy Questionnaire was completed before the intervention and after 8 weeks of the intervention.

#### 2.6.4. Home Program Self-Rating Form

The home program self-rating form was used to assess the extent to which the home program was executed and was completed by the parents of the participants. The parents were asked to complete the form when they visited the hospital every week for the early intervention program. On the home program self-rating form, the parents indicated the frequency of, the time of, their confidence in their execution skills for, and their understanding of implementing the home program. The frequency and time of performing the home program were recorded as averages for the past week. Execution skills and understanding were measured using a 5-point scale. A higher score indicated that the parent had more confidence in their own execution skills or a stronger understanding of the content of the exercise. To confirm whether the participants implemented the home program, every week, the home program self-rating form requested that the parents assess and report whether they considered anything to be difficult or notable. After 8 weeks of the intervention, semi structured interviews were conducted with each parent to understand the parents’ willingness to implement the home program and the influencing factors.

### 2.7. Informal Interviews

Qualitative data were obtained from the weekly home program self-rating form and the informal interviews conducted at the end of the 8-week intervention. The informal interviews were used to determine whether the parents were willing to continue the home programs and the factors influencing their adherence to the exercises (see Supplementary S1 and S2).

### 2.8. Home Program

This study employed the Goal Attainment Scale to set the goals for the children in the intervention group. Each therapeutic goal was rated from −2 to +2, with −2 indicating the current performance level, −1 indicating performance below expectations, 0 indicating the expected level of achievement, +1 indicating performance surpassing expectations, and +2 indicating the potential best performance. The primary intention was to establish individualized and measurable goals for the participants in the intervention group. Target achievement dates were determined through collaboration between the parents and therapists of the children, and such collaboration was also used to establish functionally meaningful goals for the family. Three objectives were formulated for each participant, and these objectives were reviewed every 4 weeks for progress, at which time discussions were completed with the parents to determine whether modifications were required (see Supplementary S3 for an example of the Goal Attainment Scale). Once the objectives were established, the therapists and parents designed a home program with consideration of the home environment. The parents were asked to engage in at least 30 min of the home program once a day, at least 5 days a week, over an 8-week intervention period while maintaining the original therapeutic program.

The comparison group continued to participate in the original therapeutic program. During posttherapy consultations, the original therapists advised the parents of the comparison group on the home program according to routine practice, without setting specific requirements regarding exercise frequency or duration. The content of the home program mainly consisted of functional training, facilitation, balance training, muscle strengthening, endurance training, and perceptual-motor coordination training.

Regarding the duration of the home program, the intervention group had a weekly average exercise time of 91.86 min per week, and the comparison group had a weekly average exercise time of 54.50 min per week. The Wilcoxon rank-sum test was used to analyze the time spent on the home program by the intervention and comparison groups. The *p* value was 0.352, which indicates the two groups did not significantly differ.

### 2.9. Data Analysis

In this study, IBM SPSS 20 software was used for statistical analysis. The chi-square test was used as a homogeneity test for the characteristics of the two groups of young children and for family characteristics. However, if the number of variable samples was less than 5, Fisher’s exact test was used as the homogeneity test for the two groups. The Wilcoxon rank-sum test was used to analyze the differences in the results of the Parent–Child Interaction Questionnaire and the Parental Self-Efficacy Questionnaire between the two groups before and after the intervention. The Wilcoxon signed-rank test was used to analyze the differences in Goal Attainment Scale scores, the Parent–Child Interaction Questionnaire, and the Parental Self-Efficacy Questionnaire after the 8-week intervention in each group. Given the small sample size and the violation of the normality assumption, a non-parametric Wilcoxon rank-sum test and Wilcoxon signed-rank test were used. A *p* value of ˂0.05 was considered significant.

Semi structured interviews and the text part of the home program self-rating form were supplementary qualitative data. The coding classification is presented in Table 1. The parents of the participants were individually coded in accordance with the order of their inclusion in the study, with the 13 parents in the comparison group being coded as A–M and the 10 parents in the comparison group coded as N–W.

## 3. Results

### 3.1. Demographics

**Figure 2:**
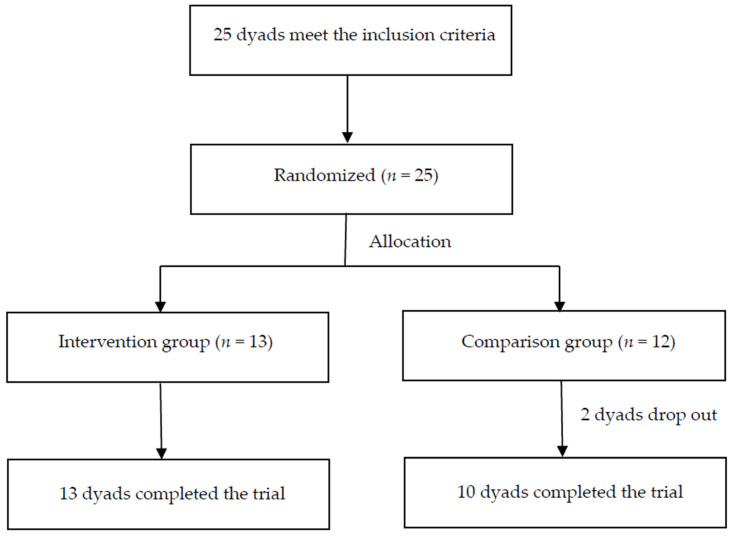
Study Flowchart.

The participants were randomly assigned to the intervention group or the comparison group by using a random number table. Thirteen groups comprised the intervention group, and 12 groups comprised the comparison group, with the individual groups consisting of the children and their main caregivers. In the first week of the research, two comparison groups dropped out due to the influence of the COVID-19 pandemic. After 8 weeks of intervention, 13 intervention groups and 10 comparison groups participated in the posttest. The study flowchart is presented in Figure 2.

In the intervention group, five children had comorbidities other than motor developmental delays: one had hydrocephalus, one had Costello syndrome, one had leukodystrophy, one had Angelman syndrome, and one had Prader–Willi syndrome. The comparison group included three children with comorbidities: one had Williams syndrome, one had attention-deficit/hyperactivity disorder, and one had emotional disorder.

In the pretest, no statistically significant differences were noted in the characteristics of the young children and caregivers between the two groups, as indicated in Table 2 and Table 3.

### 3.2. Changes in Goal Attainment Scale Scores After Parental Participation in Home Program for Children with Developmental Delays

The Goal Attainment Scale was used as a tool for goal setting in the intervention group. Each child’s therapist reviewed the goals at the end of the intervention and summed the three target posttest scores. In the intervention group, the average sum score on the pretest was −6. The average sum score on the posttest was 0.62, and the standard deviation was 1.90. The Wilcoxon signed-rank test was used to analyze the difference between the scores before and after the intervention. A significant difference was found after the intervention, *p* = 0.001, *r* = 0.885, indicating a large effect, as indicated in Table 4.

### 3.3. Parent–Child Interaction After Parental Participation in Home Programs

The differences before and after the intervention were tested using the Wilcoxon signed-rank test. No significant differences were noted between the intervention group and the comparison group with respect to psychological interaction, behavioral interaction, language interaction, and overall parent–child interaction after the 8-week intervention. No significant differences were noted between the two groups before and after intervention, as indicated in Table 5. Although the difference did not reach statistical significance, the intervention group showed a Wilcoxon *r* effect size of 0.499 for Behavioral Interaction, indicating a moderate effect that is also a practically meaningful effect.

In the interviews conducted after the 8-week intervention, the parents discussed possible factors affecting their parent–child interactions during the implementation of the home program.

### 3.4. Children’s Limited Cooperation and Parental Overwhelm in Daily Contexts

Results indicated that when children perceived exercise activities as uninteresting or overly challenging, or when they experienced frequent criticism, they were more likely to show resistance to participation. In addition, in families where everyday parent–child communication was characterized by tension or frequent conflict, physical activity sessions were sometimes described as another context in which disagreements occurred rather than as a positive shared experience. These factors may have influenced the quality of parent–child interactions observed during physical activity.

*Sometimes when I wanted the child to practice some more difficult exercises, the child resisted more and did not want to complete them.*

(20220422.I.C)

*If the child could accomplish the exercise, he would be more cooperative. But if it was too difficult for him, it would be apparent that he did not want to do it. Sometimes, a semicoercive approach, such as withholding meals or preventing him from playing with his favorite toys until the task was completed, had to be used. Typically, the child would become more willing to cooperate because they understood that not completing the task would result in deprivation of something they want. However, over time, this can affect the parent–child relationship. Nevertheless, it does help in gaining a better understanding of the child’s emotions and personality.*

(20220512.I.D)

*Because of my busy work schedule, I sometimes didn’t have time to interact with my children. I felt guilty about it, but I still made an effort to overcome this challenge.*

(20220510.I.)

### 3.5. Parental Self-Efficacy After Parental Participation in Home Programs

The within-group differences measured using the Wilcoxon signed-rank test indicated significant improvements in parenting confidence and overall parental self-efficacy within the intervention group after the 8-week intervention. Furthermore, the Wilcoxon *r* effect size were both greater than 0.7, indicating a large effect. The comparison group exhibited no significant differences in any aspect of parental self-efficacy or overall parental self-efficacy after the intervention.

The differences between the groups were analyzed using the Wilcoxon rank-sum test. At pretest, no significant differences were noted between the intervention and comparison groups for any aspect of parental self-efficacy. However, the intervention group significantly differed from the comparison group in parenting competence, parenting confidence, and overall parental self-efficacy at posttest. The intervention group scored higher than the comparison group did. Furthermore, overall parental self-efficacy at posttest, *r* = 0.629, representing a large effect, which was also clinically meaningful. The results were presented in Table 6.

The parents reported a noticeable change in themselves and their children during the follow-up interviews after 8 weeks. They observed that children were more willing to participate and engage in outdoor activities when interventions incorporated pleasant, motivating stimuli and matched the child’s preferred ways of learning. When activities were designed to support successful movement experiences, children demonstrated greater enthusiasm and confidence, which in turn encouraged parents to provide more frequent learning opportunities, such as outdoor activities.

These experiences highlight the importance of identifying each child’s preferred learning style as a foundation for effective intervention. Although developing appropriate strategies may initially require effort and experimentation, parents reported that once a suitable approach was identified, children’s learning progressed more rapidly. During the implementation of home program, parents were motivated to enhance their children’s engagement in order to facilitate cooperation, which in turn contributed to improvements in parental self-efficacy.

*When participating in outdoor activities, the children showed a stronger willingness to engage when presented with pleasant stimuli that helped them perform the correct movements. The parents also made an effort to take their children outdoors more frequently to encourage them to develop confidence.*

(20220325.W.C)

*If a child is enthusiastic about learning, parents must be willing to continue implementing such programs. However, the first step is to identify the child’s preferred learning methods. Although it can be challenging to craft effective approaches, once a suitable method is identified, the child’s learning progresses quickly. Therefore, parents must make an effort to seek methods of enhancing the child’s motivation.*

(20220526.I.H)

## 4. Discussion

This study adopted a mixed-methods design, incorporating qualitative parental interviews as a supplementary explanatory component to gain a more comprehensive understanding of the research findings. After participating in the 8-week home program for children with motor developmental delays, significant improvements were observed in the Goal Attainment Scale (GAS) scores, suggesting that children in the intervention group generally reached their targeted goals as rated by their primary therapists. However, no significant improvements were observed in parent–child interaction. Parent interviews suggested that challenges in engagement, particularly when activities did not align with children’s interests or daily routines, along with competing demands on parents’ time, may have contributed to these results.

The study also found significant improvements in parenting competence, parenting confidence, and overall parental self-efficacy. Parents reported becoming more aware of strategies to guide their children through the home program, reflecting increased confidence and competence in supporting their child’s development.

### 4.1. Improvements in Children’s Goal Attainment

The observed improvements in GAS scores in the intervention group are consistent with prior research demonstrating the benefits of family-centered interventions. For example, Almasri et al. [36] reported that parents of children with physical disabilities perceived increased family empowerment, improved child development, and greater satisfaction with services following interventions that actively involved parents. Dempsey and Keen [8] also found that early intervention programs strengthened parental self-efficacy and reduced parenting stress, emphasizing the important role of parents in supporting child development. These findings suggest that structured, parent-supported interventions can help children achieve individualized goals, highlighting the importance of family engagement in promoting developmental progress.

### 4.2. Limited Improvements in Parent–Child Interaction: Contributing Factors

In contrast to previous studies [27,28,29,30,31], the present study did not observe significant improvements in parent–child interaction after the 8-week home program. Prior research has shown that home-based interventions, such as the Promoting First Relationships program or video-feedback interventions, can enhance maternal sensitivity, responsiveness, and overall quality of parent–child interactions. The discrepancies with the current findings may be due to differences in participant age, intervention characteristics, outcome measures, or sample size. Interviews with parents further suggest that children’s engagement in activities and parental time constraints may influence the quality of interactions. Limited statistical power due to COVID-19–related recruitment challenges and age-related developmental factors may also have contributed. These results highlight that parent–child interaction may be more sensitive to contextual factors, and that additional strategies or supports may be needed to enhance interaction outcomes in older children or more complex home environments. Furthermore, as the Parent–Child Interaction Questionnaire relies primarily on parental self-reporting rather than observational measures, it may limit the detection of subtle nuances in interactional changes.

### 4.3. Improvements in Parental Self-Efficacy and Competence

Significant improvements in parental self-efficacy were observed following participation in the home program, aligning with prior research on family-centered interventions. Kristensen et al. [31] found that video-feedback interventions enhanced maternal confidence and sensitivity in parent–infant interactions. Similarly, Mas et al. [37] and Hughes-Scholes and Gavidia-Payne [38] reported that family-centered early interventions positively influenced parenting beliefs, parental self-efficacy, and overall well-being. In addition, studies on parent-implemented intervention strategies [10,39,40] demonstrated that training parents to apply structured techniques improved their competence in guiding children’s learning. These findings suggest that targeted support for parents can strengthen both parenting skills and confidence, which may indirectly support children’s developmental outcomes even when changes in parent–child interaction are not immediately observed.

### 4.4. Practical Implications

This study observed significant improvement in the Goal Attainment Scale results for the children with developmental delays in the intervention group, in which parents implemented the home program. In the context of parental participation, the GAS can be used to facilitate discussions with parents regarding setting appropriate goals for their children. Further, professionals and parents should work together to design home programs that are easy to implement and that are developed with consideration of the family environment. Parents being willing to engage in early intervention can promote the achievement of development goals for children with developmental delays.

According to the interviews with the parents, a key factor in successfully implementing a home program is improving the children’s compliance. Therefore, parents should engage in discussions with professionals on how they can enhance their skills in guiding their children through home programs. Professionals can offer relevant strategies to enhance their child’s motivation, such as providing reinforcement, integrating the home program into daily life, and setting appropriate goals. This can elevate parental efficacy and in turn enhance therapeutic outcomes for the children.

To assist parents in enhancing their proficiency in implementing home programs, a family-centered approach must be adopted, with respect, trust, and acceptance demonstrated by service providers in their communication and coordination with families of children with developmental delays—this involves establishing a strong partnership between professionals and parents. Given the significant effects of family-centered intervention on parental self-efficacy, which are comparable to children’s motor goals, parental self-efficacy should be considered an important outcome indicator in early intervention delivered by physical therapists.

### 4.5. Limitations and Future Research

The number of cases included in this study was insufficient due to the impact of the COVID-19 pandemic, and the sample size prevented the implementation of a more in-depth exploration of the research variables. Furthermore, Due to the limited sources of participants, the sample showed some comorbidity and diagnostic heterogeneity, which may affect the interpretation of the intervention effects. Future studies should increase the sample size to include more perspectives from parents to thereby enhance the depth and breadth of the research. Due to ethical considerations, all participants in this study continued to receive early intervention services, making it difficult to eliminate interference from other interventions. In addition, some participants may have been receiving concurrent interventions from other institutions, and controlling for the effects of different interventions in each case was difficult. Further, the sample of this research solely included service recipients, which might have introduced bias into the results. In the future, including both service providers and service recipients as study participants may lead to a more comprehensive understanding of the effectiveness of home programs for the parents of children with developmental delays.

One limitation of this study is that the GAS was rated by the treating therapist, which may have introduced rater bias, and it may have potential bias in therapist ratings. Although GAS is commonly used in clinical settings, future studies should consider incorporating an independent or third rater and examining inter-rater reliability to reduce potential bias and strengthen the objectivity of outcome assessment. Future studies should incorporate more objective validation methods. In addition to parental perspectives, observations from therapists could be included. For example, direct observations could be used to understand parent–child interactions, and parenting competence could be considered alongside qualitative and quantitative data. This would offer a deeper understanding of the changes that occur between parents and their children during the implementation of a home program. Further, due to limited researcher human resources, our study employed a single-blind design. For home program counseling, the children’s original therapists provided guidance. Although standardized intervention protocols were established, potential biases may have been present. Future studies should employ a double-blind design to further enhance the credibility of the research.

The questionnaire was adapted from existing literature or clinical practice, and the original instrument had established psychometric support; therefore, a full validation was not conducted in this study, which may have affected the credibility of the study results. Future studies should further establish psychometric report of the questionnaire to confirm the accuracy of the study findings.

## 5. Conclusions

The present study found that parental participation in home programs for children with motor developmental delays did not result in significant changes in parent–child interactions before and after the intervention. However, significant improvements were observed in GAS outcomes and parental self-efficacy. Despite the brief intervention period and contextual constraints during the COVID-19 pandemic, these findings provide preliminary support for the value of family-centered practices in early intervention.

The results suggest that collaboration between parents and therapists may facilitate the attainment of functional goals for young children with developmental delays and enhance parental self-efficacy, even if changes in parent–child interaction require longer interventions to become measurable. Clinically, these findings highlight the importance of supporting parental involvement in home programs and embedding intervention goals within family routines and daily contexts. The 8-week structured home program demonstrates how therapy can be effectively integrated into everyday life, bridging the gap between clinic-based treatment and home practice. Physical therapists may consider incorporating GAS as a standard tool to monitor progress and strengthen parental self-efficacy.

These findings should be interpreted with caution given the small sample size, short intervention duration, and contextual constraints, representing preliminary results. Future research using larger samples, longer follow-up, independent outcome measures, and interventions aligned with children’s interests and family contexts is warranted to further examine the effects of parental participation on child and family outcomes.

## Figures and Tables

**Table 1:** Coding Classification.

Data Categories	Code	Description
Semi structured Interview	I	e.g., 20220422.I.C represents 22 April 2022, interview with parent C
Text part of the home program self-rating form	W	e.g., 20220325.W.C represents 25 March 2022, qualitative data provided by parent C

**Table 2:** Characteristics of Young Children.

Variable		Intervention Group n = 13	Comparison Group n = 10	*p*
		M (SD)	M (SD)	
Age (years)		4.72 (1.30)	4.78 (0.72)	0.880
Sex ^a^	male	8	5	0.685
	female	5	5	
Primary Caregiver ^b^	father	1	1	0.413
	mother	12	7	
	other	0	2	
Birth order of children ^b^	only child	6	3	0.535
	the eldest	3	4	
	the second child	2	3	
	other	2	0	
Educational status of the children ^b^	not enrolled in school	2	0	0.412
	infant care center	0	0	
	kindergarten	8	9	
	other	3	1	
Early intervention programs of children ^b^	limited to our hospital	2	4	0.341
	also enrolled in other programs	11	6	
Number of siblings living together ^b^	0	6	2	0.494
	1	5	6	
	2	1	0	
	≧3	1	2	

^a^: Chi-square test. ^b^: Fisher’s exact test.

**Table 3:** Characteristics of Caregivers.

Variable		Intervention Group n = 13	Comparison Group n = 10	*p*
		*N*	*N*	
Age (years)	21–30	1	0	0.412
	31–40	10	7	
	41–50	2	1	
	51+	0	2	
Level of education	Below high school	0	1	0.426
	High school or vocational school	1	3	
	Junior college	2	1	
	Bachelor’s degree	8	3	
	Master’s degree	1	2	
	Doctorate	1	0	
Full-time primary caregiver	Yes	9	6	0.490
	No	4	4	
Annual household income (new Taiwan dollar)	Below 75,000	1	0	1.000
	75,001–112,500	2	2	
	112,501–540,000	3	2	
	540,001–1,210,000	4	3	
	1,210,000+	3	3	
Household income structure	Single income	9	6	0.490
	Dual income	4	4	

**Table 4:** Goal Attainment Scale Results for Intervention Group.

	M	SD	*Z*	*p*	*r*
Pretest	−6	0			
Posttest	0.62	1.90	−3.192	0.001	0.885

**Table 5:** Scores of Parent–Child Interaction Questionnaire.

Variable	Pretest	Posttest	Z	*p* ^a^	Wilcoxon *r*
	Median (IQR)	Median (IQR)			*r*
Psychological interaction Intervention group (n = 13) Comparison group (n = 10) *p* ^b^	44.0 (41.50–47.00) 40.5 (32.00–43.75) 0.066 ^b^	46.0 (41.00–47.00) 42.0 (34.00–47.00) 0.303 ^b^	−1.235 −0.767	0.217 ^a^ 0.443 ^a^	0.343 0.243
*r*		0.215			
Behavioral interaction Intervention group (n = 13) Comparison group (n = 10) *p* ^b^	36.0 (29.00–38.50) 32.5 (26.75–34.25) 0.128 ^b^	33.0 (31.00–36.50) 32.0 (26.50–35.00) 0.225 ^b^	−1.800 −0.103	0.857 ^a^ 0.918 ^a^	0.499 0.033
*r*		0.253			
Language interaction Intervention group (n = 13) Comparison group (n = 10) *p* ^b^	36.0 (26.50–39.00) 34.0 (26.50–39.25) 0.901 ^b^	30.0 (26.00–40.00) 35.0 (32.50–38.00) 0.641 ^b^	−0.770 −0.086	0.441 ^a^ 0.931 ^a^	0.214 0.027
*r*		0.097			
Overall interaction Intervention group (n = 13) Comparison group (n = 10) *p* ^b^	118.0 (91.00–123.00) 105.5 (85.75–113.75) 0.215 ^b^	110.0 (99.50–122.50) 109.0 (93.00–121.00) 0.576 ^b^	−0.665 −0.867	0.506 ^a^ 0.386 ^a^	0.184 0.274
*r*		0.117			

^a^: Wilcoxon signed-rank test. ^b^: Wilcoxon rank-sum test.

**Table 6:** Scores on Parental Self-Efficacy Questionnaire.

Variable	Pretest	Posttest	*Z*	*p* ^a^	Wilcoxon *r*
	Median (IQR)	Median (IQR)			*r*
Parenting competence Intervention group (n = 13) Comparison group (n = 10) *p* ^b^	32.0 (29.50–35.00) 27.50 (22.75–35.00) 0.152 ^b^	35.0 (30.00–37.00) 29.0 (24.00–33.50) 0.020 ^b^*	−1.115 −1.411	0.265 ^a^ 0.158 ^a^	0.309 0.446
*r*		0.487			
Parenting confidence Intervention group (n = 13) Comparison group (n = 10) *p* ^b^	21.0 (16.00–28.00) 21.0 (15.75–29.25) 0.975 ^b^	28.0 (27.00–30.00) 21.5 (17.50–28.25) 0.034 ^b^*	−2.594 −0.983	0.009 ^a^* 0.326 ^a^	0.719 0.311
*r*		0.442			
Self-evaluation of parenting role Intervention group (n = 13) Comparison group (n = 10) *p* ^b^	23.0 (21.00–24.00) 22.0 (16.75–23.25) 0.139 ^b^	24.0 (22.00–27.50) 23.0 (17.75–24.00) 0.142 ^b^	−1.163 −0.704	0.245 ^a^ 0.482 ^a^	0.323 0.223
*r*		0.306			
Overall parental self-efficacy Intervention group (n = 13) Comparison group (n = 10) *p* ^b^	75.0 (72.50–82.50) 70.0 (59.50–84.25) 0.153 ^b^	89.0 (82.50–91.50) 69.5 (62.75–80.75) 0.003 ^b^*	−2.833 −0.510	0.005 ^a^* 0.610 ^a^	0.786 0.161
*r*		0.629			

^a^: Wilcoxon signed-rank test. ^b^: Wilcoxon rank-sum test. * *p* < 0.05.

## Data Availability

The data that support the findings of this study are available from the first author upon reasonable request.

## Supplementary Materials

The following supporting information can be downloaded at https://www.mdpi.com/article/10.3390/children13020309/s1: Supplementary S1: Home Program Self-Rating Form (weekly completion); Supplementary S2: Home Program Self-Rating Form (complete after 8 weeks of intervention); Supplementary S3: Example of Goal Attainment Scale.
