# Supplementary material for: Enhancing Parent–Child Interaction and Self-Efficacy in Motor Skills Development for Young Children with Developmental Delays
Source: Children (Basel). 2026 Feb 23;13(2):309. doi: 10.3390/children13020309 (PMC12939759; doi:10.3390/children13020309)
Supplement: Supplementary file 1 [file children-13-00309-s001.zip › children-4101627-supplementary.pdf]

## Supplementary S1

## Home Program Self-Rating Form (weekly completion)

| | |
|---|---|
| **1. Number of days engaged in home program in the past week** | **Mon Tue Wed Thu Fri Sat Sun** |
| **2. Duration of each home program session (minutes)** | |
| **3. Self-perceived skills in performing home program** | Briefly describe your experiences |
| **4. Understanding of home program** | Briefly share your perspective |
| **5. Did you encounter any difficulties while implementing the home program in the past week?** | |
| **6. Did you experience any notable incidents while engaging in the home program over the past week?** | |

## Supplementary S2

## Home Program Self-Rating Form (complete after 8 weeks of intervention)

| | | | | | | | |
|---|---|---|---|---|---|---|---|
| **1. Number of days engaged in home program in the past week** | **Mon** | **Tue** | **Wed** | **Thu** | **Fri** | **Sat** | **Sun** |
| **2. Duration of each home program session (minutes)** | | | | | | | |
| **3. Self-perceived skills in performing home program** | Briefly describe your experiences | | | | | | |
| **4. Understanding of home program** | Briefly share your perspective | | | | | | |
| **5. Did you encounter any difficulties while performing the home program in the past week?** | | | | | | | |
| **6. Did you experience any notable incidents while engaging in the home program over the past week?** | | | | | | | |
| **7. After this study, are you willing to continue performing the home program with your child?** | | | | | | | |
| **8. What are the factors that affected your engagement in the home program?** | | | | | | | |

## Supplementary S3

## Example of Goal Attainment Scale

| Attainment Level | Score | Goal 1: Going up stairs | Goal 2: Ambulation | Goal 3: Transitions |
|---|---|---|---|---|
| **Baseline** | -2 | Ascends 6 stairs, holding the handrail, step-by-step with minimal assistance | Ambulates with walker within the classroom with minimal assistance | Transition from lying to sitting with minimal assistance |
| **Less Than Expected Outcome** | -1 | Ascends 6 stairs, holding the handrail, step-by-step under supervision | Ambulates with walker within the classroom under supervision | Transitions from lying to sitting under supervision |
| **Expected Level of Outcome** | 0 | Ascends 6 stairs, holding the handrail, step-over-step with minimal assistance | Ambulates with walker within the classroom independently | Transitions from lying to sitting independently |
| **Greater Than Expected Outcome** | +1 | Ascends 6 stairs, holding the handrail, step-over-step under supervision | Ambulates with walker to the restroom under supervision | Transitions from sitting to standing with minimal assistance |
| **Most Favorable Outcome** | +2 | Ascends 6 stairs, holding the handrail, step-over-step independently | Ambulates with walker to the restroom independently | Transitions from sitting to standing under supervision |

Name:

Target setting date:

Goal review date:
